# Extracellular Matrix in the Tumor Microenvironment and Its Impact on Cancer Therapy

**DOI:** 10.3389/fmolb.2019.00160

**Published:** 2020-01-31

**Authors:** Erik Henke, Rajender Nandigama, Süleyman Ergün

**Affiliations:** Department of Medicine, Institute of Anatomy and Cell Biology, Universität Würzburg, Würzburg, Germany

**Keywords:** extracellular matrix, cancer therapy, drug transport, immunotherapy, chemotherapy (CH), radiotherapy, tumor microenvironment, ECM

## Abstract

Solid tumors are complex organ-like structures that consist not only of tumor cells but also of vasculature, extracellular matrix (ECM), stromal, and immune cells. Often, this tumor microenvironment (TME) comprises the larger part of the overall tumor mass. Like the other components of the TME, the ECM in solid tumors differs significantly from that in normal organs. Intratumoral signaling, transport mechanisms, metabolisms, oxygenation, and immunogenicity are strongly affected if not controlled by the ECM. Exerting this regulatory control, the ECM does not only influence malignancy and growth of the tumor but also its response toward therapy. Understanding the particularities of the ECM in solid tumor is necessary to develop approaches to interfere with its negative effect. In this review, we will also highlight the current understanding of the physical, cellular, and molecular mechanisms by which the pathological tumor ECM affects the efficiency of radio-, chemo-, and immunotherapy. Finally, we will discuss the various strategies to target and modify the tumor ECM and how they could be utilized to improve response to therapy.

## Introduction

The last 25 years saw a massive shift in our approach to understand the biology of solid tumors. While research centered for a long time nearly exclusively on the individual tumor cells, the processes leading to their transformation, or conveying their malignancy, the tumor in its entirety and full complexity moved more and more the focus of cancer research. Starting from the concept of viewing the tumor as a complex organ, we meanwhile use the term tumor microenvironment (TME) to describe the entirety of the tumor components that are not malignant by themselves. Thus, the TME consists of the tumor's vasculature, connective tissue, infiltrating immune cells, and the extracellular matrix (ECM), and increasingly, all these individual components of the TME became the focus of new research communities within the fast-growing cancer field. These research efforts already resulted in the development and successful clinical implementation of TME-targeted drugs, starting with antiangiogenic agents and the potentially game-changing introduction of immunotherapeutics (Hurwitz et al., 2004; Robert et al., 2015)^1^^,^^2^. Although the ECM is probably the component of the TME that initially received the least attention, this also has changed considerably over the last decade, and numerous articles have bit by bit complemented our understanding of the tumor ECM and its role in malignancy and response to therapy.

In this article, we will focus on the impact of the ECM on the various forms of cancer therapy and on recent efforts to modulate the ECM for improved therapeutic efficacy. Of course, the ECM not only affects our efforts to treat cancer but also is directly involved in tumor establishment and disease progression. Effects of ECM-targeted approach on the efficacy of antitumor therapy often cannot be distinguished from direct effects on tumor behavior or progression. Therefore, it will be necessary to also discuss to a certain degree the immediate involvement of the ECM in malignancy and how its modification affects the course of the disease. However, to establish a base for the following review of our current efforts to include ECM targeting in the management of malignant diseases, we will first recapitulate the particularities of the ECM in solid tumors.

## Too Much, Too Disordered: The Extracellular Matrix In The Tumor Microenvironment

Many solid tumors express high levels of various ECM molecules like fibrillar collagens, fibronectin, elastin, and laminins (Provenzano et al., 2008; Mammoto et al., 2013). In addition, some cancers, that is pancreatic ductal adenocarcinomas (PDACs), are particularly rich in hyaluronan (Provenzano and Hingorani, 2013). In many tumors, the ECM compromises up to 60% of the tumor mass. Source of these ECM molecules are the tumor cells themselves, but to an even larger degree cancer-associated fibroblasts (CAFs) (Casey et al., 2009; Naba et al., 2012). Indeed, the infiltration of fibroblasts/myofibroblasts and the subsequent accumulation of significant amounts of collagenous ECM is observed in many solid tumors. This process, called desmoplasia, is strongly linked to poor prognosis and resistance to systemic therapy (Conti et al., 2008; Schober et al., 2014). By supporting tumor cells via paracrine stromal cell-derived factor-1 (SDF1) and transforming growth factor beta (TGFβ) signals, CAFs further contribute not only to a more malignant tumor phenotype by driving epithelial-to-mesenchymal transition (EMT) but also induce production of collagen and other ECM molecules (Zode et al., 2009; Porsch et al., 2013; Garcia et al., 2016). All components of the TME vary significantly from their respective counterparts in non-malignant tissues. Consequently, also the tumor ECM diverges strongly not only in amount of deposition but also in composition, organization, and post-translational modification from the ECM in surrounding normal tissue. In invasive ductal carcinomas, collagen production is shifted toward Col I and Col III compared to benign mammary lesions (Deak et al., 1991; Kauppila et al., 1998). The increased expression is mainly observed in the stromal part of the tumor. As the benign lesions also consist mainly of cells with fibroblastic characteristics, this demonstrated the tendency and capacity of breast tumor cells to shift the secreted matrisome of its stroma. In the desmoplastic stroma of breast carcinomas, up to 15% of the collagenous matrix consists of Col V, a collagen isoform of low abundance in normal and fibrocystic (<0.1%) breast tissue (Barsky et al., 1982). Experiments like second harmonics observation of fiber formation indicate that increasing the Col V/Col I ratio reduces length and organization of collagen fibers (Ajeti et al., 2011). At higher ratios, fiber formation can be completely inhibited, resulting in a gel-like ECM (Pucci-Minafra and Luparello, 1991). Col IV, on the other hand, is downregulated in ovarian carcinomas compared to benign tissue, and the expression is inversely correlated with stage and markers of malignancy (Bar et al., 2004). The same pattern of enhanced Col I/Col III expression and reduced Col IV expression is observed in human lung tumors (Fang et al., 2019). In melanoma, increased Col I expression is observed and correlated with invasiveness, angiogenesis, and reduced survival (van Kempen et al., 2008; Miskolczi et al., 2018). High collagen messenger RNA (mRNA) expression, the aberrant form of fibrous collagen spindles, and the increased expression of matrix proteases are signs of an fast turnover of the collagen in tumors (Kauppila et al., 1998).

### Collagens

The synthesis and maturation of collagens is a complex process. The increased collagen production in the TME requires not only increased transcription and translation of collagen encoding genes but also the upregulation of enzymes that are necessary for proper post-translational processing and secretion of collagen molecules. Indeed, collagen-processing enzymes like lysyl hydroxylases and prolyl hydrolases are often strongly expressed in the TME (Erler et al., 2006; Gilkes et al., 2013; Xiong et al., 2014). Lysyl oxidases are also significantly upregulated in many tumors, especially in desmoplastic cancers (Peyrol et al., 1997; Erler et al., 2006; Barry-Hamilton et al., 2010). This leads to an increased cross-linking of collagens and elastin, rendering the tumor more rigid and contributing—in combination with the already increased ECM deposition—to its palpability. The highly cross-linked collagenous matrix increases mechanical stress and focal adhesion kinase (FAK)-mediated signaling and reduces overall supply with oxygen and in the TME (Levental et al., 2009; Taylor et al., 2011; Baker et al., 2012; Rossow et al., 2018). Prolyl-4-hydroxylases (P4HAs) are ascorbic-acid-dependent enzymes that modify collagens intracellularly (reviewed in Gorres and Raines, 2010). The formation of hydroxyproline in this process is necessary to increase stability of helical collagen under physiological conditions. High P4HA expression increases intratumoral collagen deposition (Xiong et al., 2014). It is also strongly correlated with resistance to chemotherapy and reduced survival in triple-negative breast cancer patients (Xiong et al., 2018).

### Proteoglycans

Similar to collagens, proteoglycans (PGs), also constituting an important part of the ECM, require enzymes for correct production and assembly. PGs consist of a core protein, which is heavily glycosylated with longer shorter chains of glycosaminoglycans (GAGs). The attachment of the GAGs happens in the Golgi apparatus, where the core protein is translocated. Glycosyltransferases first attach a tetra saccharide to a serine of the core protein that functions as linker. Other glycosyltransferases then conjugate monosaccharides to the growing GAG chain. In addition to various glycosyltransferases, sulfotransferases and epimerases are needed for proper chain modification. The GAGs consist of repetitive disaccharide patterns. Depending on the composition of these disaccharides, GAGs are distinguished in chondroitin sulfate, dermatan sulfate, heparan sulfate, and keratan sulfate. PGs can differ significantly in molecular weight. Together with collagens—mainly Col II—PGs are the constituents of cartilage, underlining their capacity to contribute to tissue stiffness and sturdiness. In many tumors, increased levels of PGs are observed, but the story seems to be more complex as the changes in PG content between normal and malignant tissue lie mainly in shifts between various PGs often between low- and high-molecular weight PGs. Comparing prostate cancer to normal prostate tissue, Suhovskih et al. found increased expression of aggrecan and CSPG4 but reduced decorin levels (Suhovskih et al., 2013), while gastric cancers showed a doubling of overall GAG content and an increase in versican and decorin expression (Theocharis et al., 2003). Similarly, in squamous cell laryngeal carcinoma, versican and decorin are stronger represented than in normal tissue, while aggrecan is completely downregulated (Vynios et al., 2008). Brown et al. found breast carcinoma stroma to be strongly enriched in both versican and decorin expression along with Col I and fibronectin (Brown et al., 1999). In prostate cancer, decorin, lumican, and versican were strongly overexpressed, although this study relied on mRNA expression data of the core proteins; thus, conclusions about the actual PG content are difficult (Koninger et al., 2004). In peritumoral stroma of melanoma, versican is enriched compared to benign nevi (Gambichler et al., 2008). Sulfation patterns of GAGs also seem to change in cancer. Interestingly, it seems that, in colorectal cancer (CRC) and gastric cancer, a shift from 4- to 6-sulfation occurs, while in laryngeal carcinomas, the shift is reversed with more 4-sulfattion vs. 6-sulfation in normal tissue (Theocharis, 2002; Theocharis et al., 2003; Vynios et al., 2008).

### Hyaluronic Acid

Hyaluronic acid (HA) is a GAG that is not conjugated to peptides, and in contrast to the three others, it is not synthesized in the Golgi apparatus. Synthesis is performed by a family of three transmembrane glycosyltransferases, hyaluronan synthetase 1–3 (HAS1-3), which alternately conjugate gluconic acid and *N*-acetylglucosamine (reviewed in Weigel and DeAngelis, 2007; Passi et al., 2019). HASs are plasma-membrane-bound proteins, which probably are not only responsible for the synthesis but also for the extracellular export of the synthesized HA macromolecule via direct extrusion through an enzymatic pore. Some controversy exists about whether HA is exported via ABC transporters like MRP5 or MDR1 (Schulz et al., 2007). While treatment with ABC-transporter inhibitors like *S*-decylglutathione or trequinsin reduced HA production in human fibroblasts (Prehm and Schumacher, 2004), this approach using various inhibitors failed to block HA release in breast cancer cell lines that express the targeted ABC transporters (Thomas and Brown, 2010). However, while the involvement of ABC transporters in the production of HA is still debated, 4-methylumbelliferone (4-MU) inhibits HAS1–3 with some specificity and is effective in blocking HA synthesis (Nakamura et al., 1995; Kakizaki et al., 2004; Urakawa et al., 2012; Ikuta et al., 2017; Karalis et al., 2018). HA production is increased in many cancers, most notably in pancreatic carcinomas (Theocharis et al., 2000; Cheng et al., 2013) but also in breast cancers (Bertrand et al., 1992; Auvinen et al., 1997), CRC (Wang et al., 1996), prostate cancer (Lipponen et al., 2001), and even in brain tumors (Jadin et al., 2015). Stromal cells, i.e., fibroblasts, are often identified histologically as the main source of HA in the tumor, and tumor cells can increase HA synthesis in cocultured fibroblasts (Knudson et al., 1984). HA levels, and expression of HAS1–3, are correlated with poor prognosis (Bertrand et al., 1992; Ropponen et al., 1998; Auvinen et al., 2000; Zhang H. et al., 2016). HA acts as a ligand for CD44 and might thereby play a role in EMT, resulting in increased invasiveness and metastasis (Ghatak et al., 2010; Heldin et al., 2014). Expression of proteins that are thought to be cancer stem cell markers (CD90, CD133, EpCAM) are also reduced after inhibition of HA synthesis (Sukowati et al., 2019).

### Laminins

Laminins are also often stronger expressed in malignant tissue. In normal tissue, laminins are components exclusive of the basement membrane, resulting in a continuous well-delineated linear staining by immunohistology. This appearance is often distorted in tumors, or laminin appears to be ubiquitously distributed in the stromal parts of the tumors (Gusterson et al., 1982; Hand et al., 1985; Alon et al., 1986; Qiu et al., 2018). The loss of adherence to a defined basement membrane and the disruption of this basement membrane is of course a characteristic of invasive behavior. Conclusively, the increased laminin expression and aberrant distribution is correlated with poor prognosis and invasiveness, a fact that is documented since the early 1980s (Albrechtsen et al., 1981; Siegal et al., 1981). In breast cancer, laminin overexpression is generally observed (Alon et al., 1986).

Using a sophisticated and elaborated proteomics approach, Naba et al. analyzed the matrisome of human melanoma xenografts grown in severe combined immunodeficiency mice. As the differences in amino acid sequence between murine and human orthologs is sufficiently high for most proteins, the approach allowed them to distinguish the contribution of the (human) tumor cells and the (murine) stromal cells (Naba et al., 2012). The experiments revealed that some proteins are expressed exclusively by the tumor cells (e.g., Col7a1, Lama4, Lamb1) and others by the stromal cells (e.g., Col5a3, Lama2, Eln). Moreover, the ECM composition changes significantly during progression and metastasis.

### ECM Profiles as Prognostic Markers

Many cancers from the same tissue of origin can be subdivided according to the molecular expression profile. These molecular subtypes yield a lot of information about the tumor's metabolism, misregulation of survival and apoptotic pathways, presence of oncogenic drivers, and therefore the sensitivity and resistance toward different treatment modalities. Not surprisingly, ECM expression and deposition also differs significantly between molecular tumor subtypes. Breast cancers can be subdivided according to their expression status for estrogen receptor, progesterone receptor, and Her2 into luminal A (ER/PR^+^, Her2^−^), luminal B (ER/PR^+^, Her2^+^), Her2-positive (ER/PR^−^, Her2^+^), and basal-like/triple negative (triple-negative breast cancer, ER/PR^−^, Her2^−^) (Perou et al., 2000). Triple-negative breast cancer and, to a lesser extent, Her2 tumors show not only increased deposition of collagen but also enhanced invasion with CAFs (Acerbi et al., 2015; Takai et al., 2016).

The expression profile of ECM-related genes is also in many cancers a valuable prognostic factor. Bergamaschi et al. were able to divide breast cancer samples in four subgroups (ECM1–4) according to their ECM expression profile that correlated significantly with prognosis (Bergamaschi et al., 2008). Interestingly, the different expression subgroups correlated only slightly with results of histological evaluation that grouped the tumors according to the density of their appearance. A strong correlation of elevated ECM expression and poor prognosis was also found in luminal BCa (Riaz et al., 2012). In pediatric osteosarcoma, a signature of high ECM turnover was found to be highly prognostic for chemoresistance (Mintz et al., 2005) and, in gastric cancer expression of a signature containing Col1a1, Fn1, and Muc5a, was highly predictive of overall and disease-free survival (Jiang et al., 2019). Besides indicators for immune suppression, high expression of collagens Col3a1, Col4a1, and Col5a2 is correlated with poor prognosis in glioblastoma (Chen et al., 2018).

## Shielding And Nurturing: How The Ecm Protects The Tumor From Therapy

**Figure 1:**
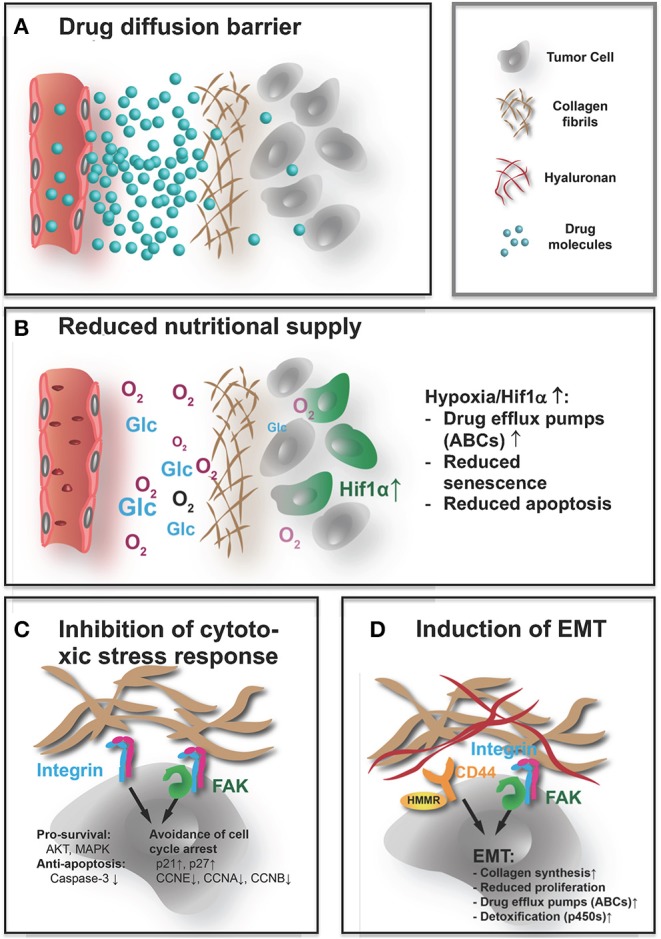
How the ECM affects the efficacy of systemic treatment. Systemically applied drugs, independently of their nature being small molecules or larger biomolecules, e.g., antibodies, peptides, or nucleic acids, have to reach their target cells and cause a therapeutic response. The abundant, highly cross-linked ECM interferes with the efficacy in both direct and indirect ways. **(A)** The rigid dense ECM acts as a diffusion barrier that impedes access of the drugs to the tumor cells, thereby acting as a shield protecting the tumor from therapeutically effective doses. **(B)** The reduced diffusion through the ECM also impairs supply with nutrients and oxygen. Pathological signaling in response to metabolic stress and hypoxia increase expression of drug efflux pumps and impair apoptosis and senescence, rendering drugs that reach the undersupplied cells less effective. **(C)** Direct contact with the ECM also affects these pathways that lead to a muted response to cytotoxic stress. Integrin and FAK activation increase prosurvival signaling, reduce apoptotic response, and help the cells to avoid cell cycle arrest when confronted with chemotherapy-induced damage. **(D)** Similarly, not only integrin and FAK but also hyaluronan induced CD44/HMMR signals can lead to EMT. The mesenchymal state is characterized by stem-like, chemoresistant traits. This includes again not only upregulation of ABC transporters and reduced proliferation but also activation of cell metabolism (cytochrome p450) that improves detoxification. That EMT also seems to increase collagen synthesis, and production of cross-linking enzymes in tumor cells might lead to a vicious cycle where the dense ECM induces EMT that again drives ECM build-up.

As we have seen, the ECM in tumors significantly differs in composition and architecture from that in normal tissue. Considering its physical properties, the tumor ECM is more abundant, denser, and stiffer. These altered characteristics can negatively affect response to therapy in multiple ways (Figure 1). Most obviously, an excessive accumulation of dense and rigid ECM, which histologically often encapsulates clusters of tumor cells, can act as a barrier, shielding the cells from therapeutic agents. This effect is directly linked to a reduced overall supply, as this barrier also impairs diffusion of oxygen, nutrients, and metabolites. Increased hypoxia and metabolic stress lead to activation of antiapoptotic and drug resistance pathways. Finally, cell–ECM contacts and increased tissue stiffness can directly contribute to chemoresistance of tumor via integrin and FAK-signaling.

### The ECM Regulates EMT and Metastasis

An aspect that is necessary to mention is meanwhile understood effect of fibrosis and CAF infiltration, and therefore also alterations in ECM composition and accumulation, on metastasis and EMT. Occurrence of metastasis imminently affects treatment options and therapeutic outcome. EMT is linked not only to increased metastasis (Yang et al., 2004; Mani et al., 2007; Ocana et al., 2012; Stankic et al., 2013) but also to chemoresistance (Singh and Settleman, 2010; Haslehurst et al., 2012; Ren et al., 2013; Fischer et al., 2015; Zheng et al., 2015; Li H. et al., 2019; Li N. et al., 2019). EMT in cancer is associated with the acquisition of a more stem-cell-like character. Accordingly, Fisher et al. found that the cyclophosphamide resistance of tumor cells that acquired a mesenchymal character could be attributed to reduced proliferation and increased expression of drug efflux pumps (ABCC1, ABCB1) and of enzymes involved in drug metabolism, like cytochrome p450s (Fischer et al., 2015), traits that are characteristic for stem and progenitor populations.

Different aspects of the ECM contribute to EMT. One critical factor is tissue stiffness. Increasing stiffness of the surrounding ECM drives EMT in breast cancer cells by promoting TWIST1 translocalization into the nucleus (Wei et al., 2015). Rice et al. found, by plating PDAC cells on substrates of different rigidity, that stiffness increases nuclear localization of the transcription factors YAP and TAZ, which can drive EMT (Rice et al., 2017). As a result, mesenchymal markers (vimentin) were upregulated, epithelial markers (E-cadherin) were reduced, and the cells demonstrated increased resistance to paclitaxel. Gemcitabine sensitivity was not affected, which was attributed to the fact that gemcitabine reversed the mesenchymal character on the stiff matrices as measured by normalized vimentin expression. Different components of the ECM might also trigger or increase EMT-like processes. The interaction of HA with hyaluronan-mediated motility receptor (HMMR) drives epicardial EMT during injury-induced heart regeneration in zebrafish (Missinato et al., 2015). In gastric cancer, the HA/HMMR axis can also be linked to EMT and 5-FU resistance (Zhang et al., 2019). *In vitro* Col I secreted by hepatic stellate cells induced EMT in hepatocarcinoma cells (Yang et al., 2014). A hallmark of EMT is the loss of epithelial polarization, which by itself is linked to anchorage of epithelial layers on a basement membrane (BM). Walter et al. found that defects in the BM and of Col IV deposition in particular can trigger EMT (Walter et al., 2018). In proximal tubular epithelial cells, Col IV helps to maintain an epithelial phenotype, while Col I promotes EMT (Zeisberg et al., 2001). Reduced Col IV synthesis or incorrect assembly and increased Col I synthesis thereby contributed to renal fibrosis. In general, the examination of the effect of collagen deposition on tumor EMT is complicated by the question of which comes first: is collagen build-up inducing EMT or are cells producing more collagen as a result of undergoing EMT. EMT is observed under pathological fibrosis in normal organs, and fibrotic collagen accumulation is often considered a result of the more mesenchymal character of the affected cells (Higgins et al., 2007; Hosper et al., 2013). This might be true for cancer, too. It has been shown that TWIST1, one of the earliest described transcription factors inducing EMT, is a potentially direct regulator of Col1a5 transcription (Garcia-Palmero et al., 2016). Similarly, the transcription factor ZEB1 positively regulates Col1 transcription and, in addition, promotes LOXL2 expression that contributes to collagen stabilization (Ponticos et al., 2004; Peng et al., 2017).

As the ECM composition within tumors itself is heterogeneous, these effects of the ECM on cell behavior and cell fate contribute strongly to tumor cell heterogeneity. In addition, there is evidence that ECM components can influence genetic instability. Deletion of the paired Col4A5 and Col4A6 genes contributes to the development of leiomyomatosis (Zhou et al., 1993). Elevated expression of MMP3 can transform cells *in vitro*, cause genomic instability in murine mammary glands, and promote carcinogenesis in these mice (Sternlicht et al., 1999; Radisky et al., 2005). Finally, blocking integrin β1 signaling attenuated tumorigenesis in an inducible human model for epidermal neoplasia (Reuter et al., 2009). Acquisition of genetic instability is an early event in tumorigenesis. As in these examples, ECM alterations drive tumorigenesis or directly genetic instability, it is likely that also later changes in ECM accumulation or composition influence genetic instability, thereby contributing to tumor progression and genetic heterogeneity.

### Effects of the ECM on Tumor Supply and Response to Chemotherapy

Distribution of drugs in the tumor occurs mainly by diffusion. Owing to lack of lymphatic drainage, convection is in most solid tumors of less importance (Welter and Rieger, 2013; Dewhirst and Secomb, 2017). An abundant and highly condensed ECM therefore can significantly reduce drug transport, resulting in only a small volume of the surrounding tissue being supplied by the individual vessels. Solid tumors are already characterized by low microvessel density (Offersen et al., 1998; da Silva et al., 2009). The often observed abundant, highly cross-linked ECM further aggravates the supply situation, leading to hypoxia and metabolic stress. Hypoxia again is directly linked to resistance vs. various forms of cytotoxic therapy and radiotherapy (Moeller and Dewhirst, 2004; Doublier et al., 2012; Jain, 2014; Horsman and Overgaard, 2016; Graham and Unger, 2018).

The family of the five lysyl oxidase isoenzymes (LOX and LOXL1–4) catalyzes the formation of cross-links between collagen molecules and in elastin networks (reviewed in Smith-Mungo and Kagan, 1998; Lucero and Kagan, 2006; Siddikuzzaman et al., 2011). They are essential for stabilization of collagen networks in a last extracellular maturation step. Thus, increased lysyl oxidase levels affect drug distribution in two ways: through better collagen stabilization toward degradation, they enhance collagen accumulation and the increasing quantity of cross-links turns the collagen network denser, further decreasing diffusivity (Rohrig et al., 2017; Rossow et al., 2018). Stromal fibroblasts contribute strongly to the elevated lysyl oxidase levels observed in many cancers (Peyrol et al., 1997). Using real-time confocal imaging of multicellular tumor spheroids, Schutze et al. demonstrated that diffusion of chemotherapeutics like doxorubicin is significantly hampered by LOX or LOXL2 overexpression (Schutze et al., 2015). Inhibition of lysyl oxidases with 2-aminopropionitril reversed the effect. Lysyl oxidases can directly confer intrinsic chemoresistance in human tumors (Rossow et al., 2018). The initially findings of this study, that lysyl oxidases are upregulated in a defined subgroup of intrinsically resistant tumors of breast, ovarian, and CRC, were substantiated by the observation that overexpression of lysyl oxidases rendered previously sensitive and lysyl oxidase–low murine tumors completely resistant to chemotherapeutic treatment. Inhibition of lysyl oxidases generally improves oxygenation (Erler et al., 2006; Rossow et al., 2018). In response, vascular endothelial growth factor A (VEGF-A) expression, the main angiogenic factor, that is a Hif-1α-regulated protein, is reduced with positive effects on tumor vascular maturation and patency (Maxwell et al., 1997; Rossow et al., 2018). Lysyl oxidases might also have a direct effect on VEGF-A expression via oxidation of the platelet-derived growth factor receptor extracellular domain (Baker et al., 2013). In addition, the increased tissue rigidity in LOXL2-high tumors facilitates endothelial invasion of the tumor tissue, a critical step in neo-vessel formation, presumably by increasing motility via FAK signaling (Zaffryar-Eilot et al., 2013).

Treatment with hyaluronidases *in vivo* reduces HA content and improves gemcitabine and DOX uptake in murine pancreatic ductal adenocarcinoma (PDAC) models (Provenzano et al., 2012; Jacobetz et al., 2013). In osteosarcoma, xenografts uptake of liposomal DOX could be improved with hyaluronidase treatment (Eikenes et al., 2005). Especially, PDACs display high hyaluronan content and can bind large amounts of water in the ECM leading to increase in interstitial fluid pressure (P_IF_). Some studies indicate that transcapillary transport and diffusion within the tumor might be hindered by high P_IF_ resulting from high HA contend and/or vessel leakage. It has to be shown if also tumors with lower hyaluronan content respond to this treatment with better drug distribution. In two of these studies, also improved vascular perfusion and reduced vessel collapse were observed after hyaluronidase treatment (Eikenes et al., 2005; Jacobetz et al., 2013). This might indicate that the high P_IF_ in hyaluronan-rich tumors restricts drug transport mainly by compressing the supplying vessels and less by interfering with interstitial drug diffusion. This would be in line with mathematical models that indicate that P_IF_ has only a minor effect on diffusion (Eikenberry, 2009).

In conclusion, it remains to be stated that a close connection exists between the signaling pathways that regulate ECM formation and angiogenesis. Especially the shared regulation via the hypoxia-response axis results in the fact that interventions that alter either the tumor ECM or the vasculature will likely also affect the other. Effects on drug response and delivery are therefore often difficult to pinpoint on a clear ECM or vascular mechanism.

### Carcinoma-Associated Fibroblasts

As carcinoma- or tumor-associated fibroblasts (CAFs) are the main source of the ECM in tumors, it is necessary to have a closer look at the particularities of these cells (Bagordakis et al., 2016; Pankova et al., 2016; Pasanen et al., 2016). CAFs are found in all solid tumors (Puram et al., 2017; Zhao et al., 2018). They differ substantially from the quiescent, metabolically inactive fibroblasts found in normal connective tissue, as they are migratory, growth and immune response promoting, and synthetically active (reviewed in Kalluri, 2016). The source of CAFs varies strongly and often according to tumor type. Stellate cells, bone-marrow-derived mesenchymal stem cells, mesenchymal stem cells from adipose tissue, and resident quiescent fibroblasts have been identified as cells of origin for CAFs (McDonald et al., 2015; Barcellos-de-Souza et al., 2016; Borriello et al., 2017; Ohlund et al., 2017). Not surprisingly, given their varying origin, CAFs are a heterogeneous cell population that can have strong differences in morphology, cell–cell interaction, and expression profile. However, they share common characteristics, as they are synthetically active, mobile, and invasive, and promote proliferation and immune response. All this is in stark contrast to the quiescent, metabolically inactive fibroblasts found in normal connective tissue (for a review on CAFs, see Kalluri, 2016).

CAFs already contribute to chemoresistance by themselves via various mechanisms: PAI-1, a cytokine produced by CAFs, activates Erk/Akt signaling and suppresses caspase-3 activation, which is necessary for tumor apoptosis following chemotherapeutic stress (Che et al., 2018). Similarly, interleukin 6 (IL6) is also produced predominantly by CAFs and induces expression of resistance-mediating CXCR7 in tumor cells (Qiao et al., 2018; Xu et al., 2018). Especially under hypoxic conditions, CAFs produce high levels of TGFβ, which induces stem cell-like properties in tumor cells including increased resistance to chemotherapy (Tang et al., 2018). Finally, the complex ECM produced by CAFs interferes with therapeutic response by forming a shielding barrier and providing prompts for protective signaling, e.g., via interaction with integrins and cadherins (Eke et al., 2010; Naci et al., 2012; McGrail et al., 2015; Jakubzig et al., 2018; Naik et al., 2018). Based on these observations, it should be possible to significantly improve drug distribution and response to therapy by depleting CAFs in the tumor stroma. However, CAFs seem to have an ambivalent role: depletion of αSMA^+^ CAFs model induced immunosuppression in a murine PDAC model and being strongly detrimental to the survival of the animals (Ozdemir et al., 2014). Thus, targeting CAFs might not be a straightforward solution. As outlined above, CAFs are a heterogeneous cell population, with in all likelihood different functions in the TME. Understanding these functions might help to separate the divers CAF populations and to develop strategies aimed at specific subgroups. Recently, it has been shown that chemoresistance is mainly driven by a population of CD10^+^GPR77^+^ CAFs (Su et al., 2018). Depletion of these CAFs with an anti-GPR77 antibody increased sensitivity to cytotoxic treatment. However, the authors pinpointed the mechanism on IL6 and IL8 that is produced specifically by CD10^+^GPR77^+^ CAFs and induces a stem-like phenotype in the cancer cells including increased chemoresistance. Whether CD10^+^GPR77^+^ CAFs also contribute disproportionally to ECM formation needs to be seen. Another idea is to reprogram CAFs back to a quiescent phenotype by inhibiting activating pathways, e.g., nuclear factor kappa B (NFκb) signaling that is increased in activated fibroblasts. Inhibition of NFκb reversed the typical CAF phenotype and improved response to cisplatin in ovarian cancer xenografts (Xu et al., 2018). The authors also showed that NFκb inhibition strongly reduced the deposition of collagen in the tumor matrix observed after cisplatin treatment. Activated CAFs also express vitamin D receptor (Sherman et al., 2014). Treatment with calcipotriol, a vitamin D analog, reversed CAFs back to stellate cells, normalized the TME, and increased gemcitabine concentration in the tumor resulting in improved response in PDAC models (Sherman et al., 2014). CAFs display an invasive and migratory phenotype that is also reflected in ROCK-pathway activation compared to quiescent fibroblasts. The ROCK inhibitor, fasudil, reduced collagen deposition, enhanced gemcitabine uptake, and improved treatment response in a transgenic PDAC model (Whatcott et al., 2017). Interestingly, the authors were able to attribute the improved treatment response to improved drug delivery after reduced collagen deposition that resulted from a reversal of the CAFs to a stellate cell phenotype.

In summary, the effects of the ECM and of CAFs on response to chemotherapy are multifaceted: forming a protective barrier that impedes drug diffusion, increased antiapoptotic effects through integrin and FAK signaling, and activation of drug resistance pathways by hypoxia and metabolic stress caused by reduced supply. In addition, ECM-targeting drugs can have toxic effects on the tumor cells themselves. Thus, an improved response to therapy after ECM targeting by itself cannot be attributed to a clear mechanism. In general, additional experiments have to be planned into the studies to prove that the ECM also directly blocks access of antineoplastic drugs to the tumor cells. The studies that were able to demonstrate a direct effect of the ECM on drug delivery and distribution within the tumor are summarized in Table 1.

**Table 1:** Effect of ECM components on tumor drug delivery.

**ECM component**	**Strategy**	**Drug**	**References**
Collagen synthesis	Fasudil treatment (ROCK inhibitor)	Gemcitabine	Whatcott et al., 2017
Collagen	Systemic treatment with collagenase	Anti-tumor antibody	Eikenes et al., 2004
Collagen	Systemic treatment with collagenase	DOX	Wang et al., 2018
Collagen	Treatment with TGFβ-inhibitor	DOX	Liu J. et al., 2012
Collagen and HA synthesis	Treatment with losartan	5-FU, DOX	Diop-Frimpong et al., 2011; Chauhan et al., 2013
Hyaluronic acid	Systemic treatment with hyaluronidase	Gemcitabine	Provenzano et al., 2012; Jacobetz et al., 2013
Hyaluronic acid	Systemic treatment with hyaluronidase	Liposomal DOX	Eikenes et al., 2005
Hyaluronic acid	HAS inhibition with 4-MU	Liposomal DOX	Kohli et al., 2014
Hyaluronic acid	HAS inhibition with 4-MU	5-FU	Yoshida et al., 2018
Lysyl oxidases	Overexpression of LOX or LOXL2, LOX(L)-inhibition with βAPN	DOX	Schutze et al., 2015

### Tumor Cell–ECM Interaction and Resistance to Chemotherapy

Cells sense their direct environment via various cell surface receptors, most notably integrins (reviewed in Kechagia et al., 2019). These surface-derived signals enable complex cellular responses to changes in ECM composition and stiffness, which may include responses that alter the cells sensitivity to therapeutics.

Already 20 years ago, it was shown that adherence to ECM via integrin β1 can protect SCLC cells from etoposide-induced apoptosis by blocking proteolytic caspase-3 activation (Sethi et al., 1999). In addition to this PI3K-mediated antiapoptotic effect, ECM–integrin β1 interaction prevents G2/M arrest in response to radiation or chemotherapy by upregulation of p21 and p27 and downregulation of cyclins A, B, and E (Hodkinson et al., 2006). The authors showed that both fibronectin as well as laminin can interact with integrin β1 and cause the protective effect. A third effect of fibronectin–integrin β1 interaction might be the upregulation of survival signals via ILK/Akt/NF-κB, as proposed by another research team that demonstrated protection vs. 5-FU-induced apoptosis in oral squamous carcinoma cells (Nakagawa et al., 2014). Cells sense tissue stiffness through signals from FAK that again cooperates with integrins. FAK signals increase pro-survival pathways like AKT and MAPK. This has been shown to confer resistance to rapamycin an mTOR inhibitor (Yoon et al., 2017). Silencing of FAK with small-interfering RNA (siRNA)/short hairpin RNA (shRNA) reinstated sensitivity to docetaxel in OvCa cells and to 5-FU in CRC cells (Halder et al., 2005; Chen et al., 2010).

Recently, Xiao et al. were able to demonstrate that resistance to therapy can have even more complex causations, involving a combination of various ECM components and their respective receptors (Xiao et al., 2019). The authors used a biomatrix model that enabled them to expose glioblastoma cells to differently modified matrixes in a 3D setting. Thereby, they found that cooperation of HA/CD44 interaction and RGD-triggered integrin α_v_ signaling increases resistance through coactivation of SRC and reduction in proapoptotic BCL-2 expression. Similarly, Nguyen et al. found that increased stiffness on a hydrogel screening platform only in cooperation with collagen-integrin β1 triggered JNK expression-mediated resistance toward sorafenib (Nguyen et al., 2014).

### Effects of the ECM on Radiotherapy

Radiotherapy is considered as one of the potentially curative modalities for cancer. However, preclinical studies suggested that tumor ECM might play a pivotal role in resistance and recurrences to radiotherapy in different cancers (Cordes and Meineke, 2003; Sandfort et al., 2007; Ou et al., 2012). Tumor hypoxia or increased inflammation in TME modifies tumor ECM components and increases collagen deposition, ECM density, and stiffness (Hui and Chen, 2015; Willumsen et al., 2018). In addition, it is known that adhesion to the dense ECM modifies radiation sensitivity of cancer cells (Onoda et al., 1992).

Not only cell adhesion to the ECM but also ECM-induced signaling is mediated largely by integrins, a family of heterodimeric cell surface receptors that directly interact with the ECM (reviewed in Desgrosellier and Cheresh, 2010; Hamidi and Ivaska, 2018). Integrins play a crucial role in cell survival, proliferation, morphogenesis, tumorigenesis, and angiogenesis (Aumailley and Gayraud, 1998; Hapke et al., 2003; Reginato et al., 2003; Demircioglu and Hodivala-Dilke, 2016). Cell survival by integrins is mediated by several signaling pathways such as FAK, Src kinases, PI3K/Akt, MAP kinase signaling, NFκB signaling, p130cas/paxillin, and upregulation of Bcl2-family antiapoptotic proteins (Uhm et al., 1999; Damiano et al., 2001; Cordes et al., 2006; Hehlgans et al., 2007; Serebriiskii et al., 2008). It has also been shown that by altering integrin expression and increasing secretion of survival-promoting ECM molecules, tumor cells develop resistance to anoikis and improve survival in inappropriate ECM environments (Khwaja et al., 1997; Gilmore, 2005). Given their role in cancer cell survival, it is not surprising that integrins, and therefore the ECM-molecules they interact with, are also strongly involved in regulating resistance to radiotherapy: recent studies reported that β1 integrins are upregulated after radiotherapy and play a role in mediating resistance to radiotherapy (Onoda et al., 1992). Experiments in melanoma and sarcoma cells showed that downregulation of the p53 tumor suppressor gene resulted in increased survival following cell detachment and resistance to apoptosis (Lewis et al., 2002; Hazlehurst et al., 2003). Blocking ECM-induced signaling by targeting either β1 integrins or downstream PI3/AKT signaling enhanced the efficacy of radiotherapy in cultured breast cancer cells and implanted BCa xenografts (Liang et al., 2003; Park et al., 2006, 2008). Another study performed on A549 lung cancer cells demonstrated that cell adhesion to the ECM protein fibronectin promotes resistance to radiotherapy (Cordes and Beinke, 2004). The protective function of fibronectin is mediated by α5β1 integrin, as shown in A549 and H1299 lung cancer cells, where the increased fibronectin synthesis after cetuximab treatment attenuated cytotoxic and radio sensitivity (Eke et al., 2013). Similarly, in matrigel-embedded 3D cultures of human malignant human breast cancer cells targeting the interaction between fibronectin and α5β1 integrin enhanced radioresponse by promoting apoptosis (Nam et al., 2010). Further studies also revealed that integrin α5β3 is upregulated after radiotherapy and that treatment with an α5β3 antagonist enhanced radiosensitivity (Abdollahi et al., 2005).

Matrix metalloproteinases (MMPs) are involved in turnover and modulation of ECM components. The MMP-caused fragmentation of ECM components observed in many cancers in TME is generally associated with poor prognosis (Noel et al., 2012). *In vitro* studies have demonstrated that increased MMP activity and ECM proteolysis leads to enhanced migration, angiogenesis, and metastasis after radiotherapy, and breast cancer cells showed increased invasion capacity with increased expression of MMP2-activating molecules MT1-MMP and TIMP-2 (Paquette et al., 2007; Artacho-Cordon et al., 2012). MMPs also promote angiogenesis by degradation of ECM components and basement membrane, which furthermore can affect outcome of radiotherapy in particular by enhancing escape from stress after the treatment (Nambiar et al., 2015). Furthermore, in preclinical studies, pretreatment with MMP2 inhibitors enhanced sensitivity to radiotherapy (Qian et al., 2002; Kaliski et al., 2005; Badiga et al., 2011). Similarly, MT1-MMP blockade in murine breast carcinomas with a neutralizing antibody enhanced radiosensitivity via increased tumor perfusion (Ager et al., 2015). Interestingly, this was likely caused by a shift from proangiogenic M2 to a phagocytic M1 macrophage phenotype. Other studies also reported that NF-κB causes resistance to radiotherapy by inducing MMP2/9, which promote tumor metastasis and invasion. These studies also showed that treatment of CRC cells with nafamostat mesilate (FUT175), a synthetic serine protease inhibitor, results in downregulation of NF-κB and enhances sensitivity to radiotherapy by inhibiting MMP2/9 (Sugano et al., 2018).

TGFβ signaling modulates the TME by stimulation of myofibroblasts and other stromal cells and by increasing collagen cross-linking enzymes, particularly lysyl oxidases (Egeblad et al., 2010). Both TGFβ and lysyl oxidases can be targeted to improve response to radiotherapy: inhibition of TGFβ signaling enhances radiation sensitivity of non-small-cell lung cancer (NSCLC) cells *in vitro* and in a Lewis lung carcinoma mouse model (Kirshner et al., 2006; Du et al., 2015; Zhao et al., 2016). While radiotherapy increases LOX secretion in several tumor cell types and in *in vivo* lung adenocarcinoma xenograft models (Shen et al., 2014), knockdown of LOX2 in DU145 prostate cancer cells using a siRNA approach enhanced their radiosensitivity not only *in vitro* but also in a xenograft model (Xie et al., 2019). P4HA is another enzyme necessary for correct collagen deposition and directly responsible for increased collagen deposition in tumors (Xiong et al., 2014). Its expression is strongly correlated with response to radiotherapy in breast cancer patients (Toss et al., 2018).

The effect of ionizing radiation on cells is also strongly dependent on their oxygenation status. Hypoxia significantly impairs the effectiveness of radiotherapy (reviewed in Griffioen et al., 2001; Horsman and Overgaard, 2016; Graham and Unger, 2018). Consequently, strategies to improve oxygenation status, in tumors before radiotherapy—e.g., by pretreatment with antiangiogenic drugs—have been devised and tested. Although antiangiogenic drugs were initially designed to reduce tumor growth by starving it, there might be a period after administration when antiangiogenics briefly improve supply by impairing non-functional vessel formation (Claes and Leenders, 2008). Dings et al. found that treatment with Avastin or the antiangiogenic peptide anginex improved oxygenation in various murine tumor models (Griffioen et al., 2001; Dings et al., 2007). Scheduling radiation to line up with this improved oxygenation window enhanced the efficacy of radiotherapy in these models. Correspondingly, response to radiotherapy increased in murine tumors during the improved oxygenation observed 2 days after treatment with the VEGF-R2-inhibitor sunitinib (Matsumoto et al., 2011). However, some evidence suggests that the protective effect of hypoxia on radiation damage is at least in part mediated by Hif1α controlled release of proangiogenic and endothelial protective cytokines that restrict radiation damage on the tumor vessels (Moeller and Dewhirst, 2004). Thus, the beneficial effect of antiangiogenic therapy on response to radiotherapy might not stem from reduced hypoxia but from increased sensitivity of the growth factor signaling-deprived endothelium. Moreover, antiangiogenic treatment leads, in most settings, not to improved supply but to increased hypoxia, both in murine models and in patients (Henke et al., 2007; Keunen et al., 2011; Van der Veldt et al., 2012; Miyazaki et al., 2014; Rohrig et al., 2017). Interestingly, Riesterer et al. showed that fractionated radiation reversed the increased hypoxia after Vatalinib treatment (Riesterer et al., 2006).

### Effects of the ECM on Immunotherapy

Cancer immunotherapy is a promising concept that yielded impressive breakthroughs in recent years. The term immunotherapy is used for a variety of therapeutic approaches that all aim to engage the patient's immune system against cancer. Adoptive transfer methods are based on patient-derived lymphocytes that are expanded, genetically modified, or activated *ex vivo* before being reinfused (Robbins et al., 2015; Lu et al., 2017). In addition, the application of antitumor vaccination approaches have made considerable progress in recent years (reviewed in Rammensee and Singh-Jasuja, 2013; Accolla et al., 2019; Peng et al., 2019). The method most widely established in the clinic is the treatment with checkpoint inhibitors. Many tumor cells express ligands to T-cell receptors that, upon engagement, block immune surveillance. These ligand/receptor interactions act as inhibitory checkpoints for the adaptive immune system to prevent indiscriminate attacks on the hosts own cells. Currently, the interactions of the ligands CD80/CD86 with the receptor CTLA4 and the PD-L1 with its receptor programmed cell death receptor (PD-1) are of the most importance in cancer immunotherapy. The therapeutic success that draw so much attention in recent years can be mainly attributed to the clinical introduction of inhibitory antibodies against CTLA4 and PD-L1/PD-1 (Eggermont et al., 2018; Gandhi et al., 2018; Paz-Ares et al., 2018)^3^ As a detailed discussion of the different immunotherapeutic approaches, the regulatory pathways, and various T-lymphocyte populations involved in cancer immune surveillance is beyond the scope of this article, we want to refer the reader to the many excellent review articles that focus on these topics (e.g., Lim et al., 2018; Sharpe and Pauken, 2018; Ganesh et al., 2019).

**Figure 2:**
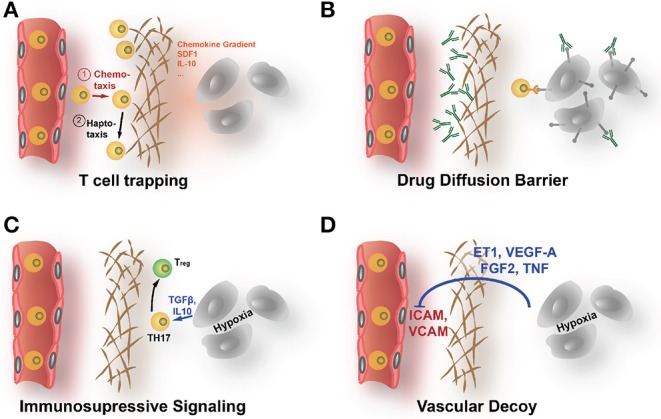
How the ECM affects the efficacy of immunotherapy. **(A)** The dense ECM can prevent immune cells to reach the tumor cells even in highly immunogenic cancers. Upon contact with areas of increased stiffness, lymphocytes are prone to follow less a chemoattractive gradient but to migrate along the fields of elevated rigidity (haptotaxis). **(B)** The shielding diffusion barrier that the ECM forms prevents also immunotherapeutic drugs, like checkpoint inhibitory ABs, to reach the tumor. **(C)** The increased hypoxia that results from poor supply behind the diffusion barrier can directly enhance immune escape by upregulation of immunomodulatory factors like IL-10 or TGF-β. **(D)** Hypoxia also increases angiogenic signals. Activated blood vessels show reduced ICAM1 expression, impeding attachment and extravasation of immune cells.

While some cancers, e.g., melanoma or NSCLC, respond well to immunotherapy and checkpoint inhibition in particular (Robert et al., 2015; Gandhi et al., 2018; Paz-Ares et al., 2018), results in other cancers, e.g., breast carcinomas or PDACs, are less striking (McArthur et al., 2016; Parra et al., 2017; Adams et al., 2018; Rugo et al., 2018). Immunotherapeutic approaches need to get both the drug and T lymphocytes deep into the tumor and in contact with the tumor cells to be effective. A major obstacle for the successful application of the immune therapeutics in some cancer patients seem to be the low infiltration with T lymphocytes, as infiltration rate is highly predictive of response (Issa-Nummer et al., 2013). The infiltration rate is not only determined by the degree the malignant cells are able to provoke an immune response (hence the tumor's immunogenicity) but also by the ECM that can act as a protective shield. Immune cells that are first attracted to side of tumor growth by cytokine gradients (chemotaxis) are often diverted from this direction when confronted with the rigid, ECM-rich encapsulation around the tumor cell clusters. The immune cells migrate then along the gradient of increasing rigidity and ECM-provided adhesion sites (haptotaxis), being diverted from the tumor cells (see Figure 2 for a detailed representation of various ways the ECM might affect immunotherapy). Thereby, the high density of the tumor ECM strongly determines not only distribution of immunomodulatory drugs but also infiltration of immune cells into the tumor (Hallmann et al., 2015; Raave et al., 2018). Increased hypoxia and metabolic stress that are in part a result of the poor diffusion in ECM-rich tumors lead to an upregulation of immunosuppressive factors like IL-10, CCL18, CCL22, TGFβ, and prostaglandin E2 and also VEGF-A (Wei et al., 2011; Xue and Shah, 2013; Schaaf et al., 2018). Especially TGFβ acts in the TME as a suppressor of infiltrating CD8^+^-cytotoxic lymphocytes (CTLs) and natural killer (NK) cells. TGFβ exercises this effect by attracting regulatory T cells (T_regs_) and by working as a M2-polarizing agent for macrophages (Φ) (Ostroukhova et al., 2006; Zhang F. et al., 2016; Schaaf et al., 2018). Both M2-Φ and T_regs_ negatively regulate infiltration and activity of CD8^+^-CTLs (Ruella et al., 2017). VEGF-A also is able to recruit T_regs_ that express NRP1, a VEGF coreceptor, and can directly suppresses activation of T cells (Gavalas et al., 2012; Powell et al., 2018). Consequently, tumor ECM remodeling, stabilization, and accumulation are increasingly recognized as crucial factors controlling infiltration and also differentiation, activation, and polarization of immune cells in the TME (Mushtaq et al., 2018). For the distribution of immunomodulatory drugs, the same consideration apply as for other antineoplastic drugs. Many immunomodulatory drugs, meanwhile, in the clinic are therapeutic antibodies, e.g., the CTLA-4 directed ipilimumab and PD-1 directed pembrolizumab (Robert et al., 2015; Adams et al., 2018; Hodi et al., 2018). Because of their large hydrodynamic diameter, diffusion of these macromolecular drugs is even more affected by a dense, strongly cross-linked ECM.

Neither T cells nor dendritic cells are able to penetrate dense fibrils in tumor ECM. Salmon et al. showed in human lung tumor specimen that the migration and finally distribution of T cells is dictated by the aligned collagen fibers surrounding tumor islets and perivascular regions in the tumor stroma (Salmon and Donnadieu, 2012; Salmon et al., 2012). This resulted in an accumulation of T cells in the stroma, where most of these immune cells got trapped without being able to reach the targeted tumor cells for destruction. Collagenase treatment ameliorated the trapping effect and increased infiltration into the areas of malignant cells. Another study in PDAC suggested that hyaluronan, by forming dense architecture, impedes infiltration of effector immune cells and drugs in a similar manner as collagen (Jacobetz et al., 2013). The clinical relevance of this shielding function of the stromal ECM that keeps immune cells at distance from the tumor cells was most strikingly demonstrated by Mariathasan et al. that showed in a urothelial cancer patient cohort that non-response to PD-L1 checkpoint inhibition correlated with CTL entrapment in the stromal ECM (Mariathasan et al., 2018).

Hypoxia in the TME, which is partially caused by the dense ECM, leads to an upregulation of angiogenic factors like VEGF. This also impairs infiltration with CTLs as endothelial cells downregulate in response to VEGF cell surface glycoproteins, such as selectins and cell adhesion molecules like ICAM1, ICAM2, and VCAM1 (Griffioen et al., 1996; Achen et al., 2005; Castermans and Griffioen, 2007; Lund and Swartz, 2010). This results in a masking of the supplying blood vessels, as the CTLs cannot longer attach to the endothelium void of the necessary adhesion proteins. Other studies found that the constantly activated tumor endothelium selectively promote transmigration of immunosuppressive T_regs_ by upregulating adhesion molecules like MadCAM1, CD62-E, CD166, and stabilin1 (Nummer et al., 2007; Shetty et al., 2011). Voron et al. reported that VEGF-A promotes immune escape by inducing T_regs_ and causing PD-1 expression on VEGF-R2 expressing CD8^+^ T cells (Voron et al., 2015). CAFs, which regulate stromal matrix and serve as a primary source for matrix-associated proteins, also play an essential role in the infiltration of leukocytes into the tumor (reviewed in Turley et al., 2015).

ECM proteins collagens, laminin, and fibronectin regulate polarization and activation of immune cells in TME (Vaday and Lider, 2000; Simon and Bromberg, 2017; Mushtaq et al., 2018). Transmembrane collagens like Col XVII can trigger immune inhibitory signaling in NK cells via leukocyte associated Ig-like receptor-1 as has be shown in multiple cell lines (Rygiel et al., 2011). In cell culture, high molecular weight HA (in contrast to low molecular weight HA) suppress the immune system by increasing activity of T_regs_, presumably acting as an TLR ligand (Bollyky et al., 2009).

MMP and ADAM metalloproteinases also modulate immune and inflammatory responses by degradation of the ECM. The ECM acts as a reservoir of immunomodulatory cytokines and growth factors that are released upon its proteolytic degradation. In addition, the cleavage products of the ECM (e.g., matrikines) can, by themselves, affect immune surveillance. Lastly, metalloproteinases are involved in release of immunoactive factors from the cell surface. ADAM10, ADAM17, and MMP9, for example, are responsible for shedding of major histocompatibility complex class I chain-related molecule A (MICA) from tumor cells (Waldhauer et al., 2008; Chitadze et al., 2013). MICA is a surface ligand and activates the immunoreceptor NKG2D, effectively marking MICA-expressing cells for elimination. Although many cancers express MICA, the upregulation of metalloproteinases enables the tumors cells to escape immune surveillance as has been shown in human prostate cancer, breast cancer, and osteosarcoma cells (Barsoum et al., 2011; Sun et al., 2011). A further link to the ECM exists in the form that ADAM10 is hypoxia regulated, that itself is increased by ECM accumulation (Barsoum et al., 2011). Enzymatic proteolysis of versican results in the release of the matrikine versikine that triggers the generation of conventional dendritic cell in CRC, which subsequently promotes T-cell infiltration (Hope et al., 2017). An immune-enhancing quality of versikine was also detected in myeloma (Hope et al., 2016). Here, however, versikine induced production of inflammatory IL1β and IL6 from myeloma-associated macrophages that increased infiltration of CD8^+^ T cells.

The ECM also directly regulates escape mechanisms, e.g., the expression of checkpoint molecules. Inhibition of HA synthesis by 4-MU in a mesothelioma xenograft lead to a significant increase in both PD-1 and PD-L1 expression (Cho et al., 2017).

Tumor-associated macrophages are the most frequent immune cells found in the TME (Lewis and Pollard, 2006). TAMs play an important role in mediating adaptive immune response in cancer. Interestingly, macrophages can have both anti- and proinflammatory roles, which is often linked to their M1 or M2 polarization status (reviewed in Tariq et al., 2017). Various ECM components are involved in TAM polarization: At least in cell culture, HA alone is able to strongly drive macrophage polarization toward a protumorigenic and anti-inflammatory M2 phenotype (Kim et al., 2019). This effect of ECM components on macrophage polarization was reported already much earlier for Col I that also drives M2 polarization (Kaplan, 1983; Wesley et al., 1998). Conversely, fibronectin-rich ECM strongly enhances the cytotoxic activity of macrophages toward tumor cells, consistent with M1 polarization (Perri et al., 1982). Although it has not been explicitly shown that ECM targeting affects immune surveillance and the response to immunotherapy by changing TAM polarization, the clear connection between the ECM and macrophage polarization, on the one hand, and macrophage polarization and immune escape, on the other, strongly imply the possibility of such an effect.

## Braking Down The Barriers: Destabilizing The Tumor Ecm To Improve Treatment Response

### Preclinical Studies

Considering the wide and negative effects of the abundant and pathologically altered tumor ECM on various treatment modalities, the interest in targeting the ECM to improve therapeutic efficacy is evident. Of course, removing the treatment-impeding ECM *in situ* is a major challenge. Nevertheless, in preclinical models, the ECM degradation has been shown to improve drug uptake and response (Table 2). For example, Eikenes et al. studied the possibility of enzymatic hydrolysis of collagen *in vivo*: Treatment of osteosarcoma xenografts with systemically injected collagenase increased uptake of an antibody specific for the implanted tumor (Eikenes et al., 2004). The collagenase treatment improved overall accumulation of the therapeutic antibody and resulted in a more homogeneous distribution. Wang et al. immobilized collagenase on nanogels and used it to treat a hepatocellular mouse model, in which it improved distribution and response to doxorubicin (Wang et al., 2018). Similarly, hyaluronidase was used to treat hyaluronan-rich tumors and to increase uptake and efficacy of gemcitabine and DOX in experimental PDAC (Provenzano et al., 2012; Jacobetz et al., 2013) and of liposomal DOX in osteosarcoma xenografts (Eikenes et al., 2005). Currently, several clinical trials with pegylated hyaluronidase (PEGPH20) are ongoing (see below).

**Table 2:** Overview on preclinical and cell culture approaches to improve response to therapy by targeting the ECM.

**ECM-targeting strategy**	**Therapeutic used in combination**	**Test model**	**References**
Collagenase	Model-specific AB^a^	Osteosarcoma xenograft in mice	Eikenes et al., 2004
Immobilized collagenase	Doxorubicin	Hepatocellular allografts in mice	Wang et al., 2018
Lysyl oxidase inhibition (2-aminopropionitril)	Doxorubicin	4T1 and EMT6 Breast cancer allografts in mice	Rossow et al., 2018
	Cisplatin	LLC allografts in mice	Rossow et al., 2018
P4HA inhibition (shRNA and Ethyl-3,4-dihydroxybenzoic acid)	Docetaxel	Breast cancer xenografts	Xiong et al., 2018
	Docetaxel and doxorubicin	Breast cancer 3D spheroids in cell culture	Xiong et al., 2018
Hyaluronidase, pegylated, i.v.	Doxorubicin	Spontaneous PDAC mouse model KPC	Jacobetz et al., 2013
	Gemcitabine	Spontaneous PDAC mouse model KPC	Provenzano et al., 2012
Hyaluronidase, intratumoral	Doxil^b^	Osteosarcoma xenograft in mice	Eikenes et al., 2005
Hyaluron synthase-inhibitor [4-methylumbelliferone (4-MU)]	Doxil^b^	4T1 Breast cancer allograft in mice	Kohli et al., 2014
	5-Fluorouracil	PDAC xenografts	Yoshida et al., 2018
	Doxorubicin	CML cells in cell culture	Uchakina et al., 2016
TGFβ-inhibition (sTβRII and anti-TGFβ-AB^a^)	Doxorubicin	4T1 Breast cancer allograft in mice	Liu J. et al., 2012
Hif-1α-siRNA	Doxorubicin	Prostate cancer xenografts	Liu X.Q. et al., 2012
Hif-1α-shRNA	Cisplatin	Prostate cancer xenografts	Gu et al., 2017
Antifibrotic drug (Pirfenidone)	Gemcitabine	PDAC xenografts	Kozono et al., 2013
	Radiation + sunitinib	LLC allografts	Choi et al., 2015
	Doxorubicin		Giri et al., 2004
Antifibrotic drug (Ormeloxifene)	Gemcitabine	PDAC xenografts	Khan et al., 2015
Antifibrotic drug (Losartan)	5-Fluorouracil		Chauhan et al., 2013
	Doxil^b^	Pancreatic adenosquamous carcinoma xenografts in mice	Diop-Frimpong et al., 2011
	Liposomal paclitaxel	Breast cancer allograft in mice	Zhang F. et al., 2016
CAF reprogramming (NFκb-inh.: metformin)	Cisplatin	Ovarian cancer xenografts	Xu et al., 2018
CAF reprogramming (Calcipotriol)	Gemcitabine	PDAC xenograft model	Sherman et al., 2014
CAF reprogramming (ROCK-Inh.: fasudil)			Whatcott et al., 2017
GRP77+ CAF depletion (GRP77–AB^a^)	Docetaxel	Patient derived breast cancer xenografts	Su et al., 2018
Hyaluronidase, intratumoral	Immunotherapy (shPD-L1 loaded nanoparticles)	B16 F1 Melanoma allografts	Guan et al., 2019
	Immunotherapy (Ovalbumin/CpG loaded nanoparticles)	B16 F1 Melanoma allografts	Guan et al., 2018
Hyaluron synthase-inhibitor (4-MU)	Immunotherapy (IL-12) + cyclophosphamide	CRC allografts in mice	Malvicini et al., 2015
TGFβ inhibition (anti-TGFβ-AB^a^)	Immunotherapy (anti-PD-L1-AB^a^)	EMT6 Breast cancer allograft in mice	Mariathasan et al., 2018
Hyaluron synthase inhibitor (4-MU)	Radiotherapy	Fibrosarcoma cell culture	Saga et al., 2017
LOXL2 inhibition (shRNA)	Radiotherapy	DU145 prostate cancer xenografts	Xie et al., 2019
TGFβ inhibition (SB431542)	Radiotherapy	LLC allografts in mice	Zhao et al., 2016
Antifibrotic drug (Pirfenidone)	Radiation + sunitinib	LLC allografts	Choi et al., 2015

a *AB: antibody*.

b *Doxil: liposomal doxorubicin*.

However, as the ECM in tumors is constantly remodeled, it might not be necessary to remove already existent ECM. The perpetual turnover is signified by concomitantly high-level synthesis of ECM macromolecules and degradation of the ECM by tumor-secreted hydrolytic enzymes like MMPs and cathepsins, on the other hand. This opens the possibility to shift the turnover toward net degradation by blocking *de novo* synthesis. This can be accomplished by either reducing cues that lead to increased expression of ECM molecules, like TGFβ signaling or hypoxia-response pathways, or by inhibiting the various modifying enzymes necessary for proper production, secretion, and maturation of these ECM molecules.

### Targeting Collagen Synthesis and Maturation

The complex post-translational modifications that collagens have to be subjected to for proper assembly, secretion, and extracellular maturation offer multiple possibilities to interfere with their accumulation in the TME. These modifications are catalyzed by enzymes that are preferred subjects for targeting with small molecule drugs.

Inhibition of lysyl oxidases reduces tissue stiffness on overall collagen deposition, as the maturation process in the form of LOX-induced cross-links further stabilizes collagens and protects them from degradation. Treatment with 2-aminopropionitrile reduced significantly collagen deposition and drug accumulation in various murine allograft models (Rossow et al., 2018). In breast cancer allografts, this leads to increased sensitivity toward DOX and improved efficacy against metastatic disease. In Lewis lung carcinomas, it improved response to cisplatin. Post-translational hydroxylation by collagen P4HA is necessary for correct intracellular processing of collagen molecules. Xiong et al. showed that inhibition of P4HA1 significantly reduced collagen deposition in breast cancer xenografts (Xiong et al., 2018). While inhibition of P4HA1 in these xenografts either with shRNAs or ethyl-3,4-dihydroxybenzoic acid had no antitumor effect on itself, it significantly improved response to docetaxel in reducing tumor growth and pulmonal metastasis. Latest clinical data might underline the necessity to conduct further studies. Recently, phase II trials combining treatment with a LOXL2-directed antibody (simtuzumab) with chemotherapy—gemcitabine in PDAC and FOLFIRI regiment in CRC—ended with unsatisfactory results (Benson et al., 2017; Hecht et al., 2017). Drug scheduling in these trials were not designed to synergistically profit from improved drug delivery after ECM modification, but on the assumption of an additive effect of the cytotoxic drugs with the antitumoral effect that has been demonstrated preclinically for LOXL2 blockade by itself (Barry-Hamilton et al., 2010; Rodriguez et al., 2010). If, as indicated in preclinical tests, the improved drug distribution after collagen destabilization is indeed a deciding mechanism by which LOX targeting can improve treatment outcome, this has to be reflected in the trial design. In patients, the changes of the ECM would take much longer to manifest, than in the generally much faster growing murine test models. Another problem in targeting lysyl oxidases is the shared substrate spectrum and the completely redundant biological activity of the five family members (Molnar et al., 2003). Targeting LOXL2 with an inhibiting antibody might be too specific, and using a small molecule drug that equally inhibits all five lysyl oxidases might yield better results. Incidentally, simtuzumab did not fare well as a stand-alone therapeutic in the treatment for various fibrotic diseases either (Raghu et al., 2017; Verstovsek et al., 2017; Harrison et al., 2018).

### Targeting Hyaluronan Synthesis

As discussed above, hyaluronan synthases (HAS1-3) can be inhibited by 4-MU. Treatment with 4-MU significantly reduces HA accumulation in various murine tumor models. This already confers antiproliferative, proapoptotic, and antimetastatic effects in cultured tumor cells and implanted tumors (Yates et al., 2015; Nagase et al., 2017). The antitumor effect results from reduced CD44 activation that lowers PI3K signaling and AKT and ERK phosphorylation (Kundu et al., 2013; Lompardia et al., 2017). Others have shown that 4-MU increases p38 activation and caspase-3, caspase-9, and PARP cleavage, explaining not only the apoptotic effect but might also indicate an increased sensitivity toward cytotoxic stress (Lokeshwar et al., 2010; Uchakina et al., 2016). Importantly, these proapoptotic effects can be rescued by HA addition to cultured cells, demonstrating that these are direct results of reduction in HAS activity and not off-target effects. The intrinsic antitumor properties of 4-MU complicate concluding whether HAS inhibition improves therapeutic response in cotreatment studies with chemotherapeutics and other tumor-cell-targeted drugs. However, several studies also looked on drug distribution and accumulation. Kohli et al. used liposome-encapsulated 4-MU to treat 4T1 allografts, resulting not only in HA reduction but also in a more heterogeneous distribution of Doxil (liposomal DOX) and reduced tumor growth in the combination treatment group (Kohli et al., 2014). 4-MU increased intracellular accumulation of fluorouracil, indicating an influence on either cell permeability or drug efflux and improved response to 5-fluorouracil in PDAC xenografts (Yoshida et al., 2018).

HA targeting also can improve immunotherapy. 4-MU treatment in syngeneic C26 CRC allografts improved infiltration of T lymphocytes and subsequent therapy with a combination of cyclophosphamide and adenovirally delivered IL12 (Malvicini et al., 2015). Intratumoral application of hyaluronidase improved response to PD-L1-directed shRNA-based immunotherapy and vaccination with ovalbumin/CpG in a melanoma mouse model (Guan et al., 2018, 2019). The authors attributed the improved response to both immunotherapy modalities to an increased T-cell infiltration rate after the hyaluronidase treatment. A positive effect of 4-MU treatment was also reported on the sensitivity of fibrosarcoma cells to radiotherapy (Saga et al., 2017). Under the trade name Hychromone, 4-MU is already marketed in Asia and Europe as a choleretic agent (reviewed in Kudo et al., 2017). As clinical experience with this drug already exists, application in a different cancer treatment setting could be probably enhanced.

### Targeting TGFβ and Hif1α

A further alternative to targeting either ECM molecules or the enzymes necessary for their production can be to interfere with the signaling pathways that lead to their upregulation in the first place. This might be an interesting strategy, as at least some of these pathways seem to be master regulators of many pathological alterations in the TME. TGFβ might be one of these key factors. TGFβ signaling has been shown to induce production of collagen, lysyl oxidases, and hyaluronan (Zode et al., 2009; Voloshenyuk et al., 2011; Porsch et al., 2013; Xie et al., 2013; Garcia et al., 2016). Treatment of murine syngeneic 4T1 breast carcinomas with a TGFβ inhibitor reduced Col I content and improved accumulation and distribution of DOX (Liu J. et al., 2012). After, establishing that TGFβ expression was a prognostic factor for the response of urothelial cancer patients to PD-L1 directed immunotherapy and that TGFβ correlated with entrapment of CD8^+^ CTLs in the stroma of the non-responsive tumors, Mariathasan et al. showed that concomitant TGFβ inhibition strongly improved CTL infiltration and outcome of checkpoint therapy in mouse models (Mariathasan et al., 2018). Another signaling pathway that affects ECM synthesis is the hypoxia response via Hif-1α-stabilization. Hif-1 signaling induces collagen expression and lysyl oxidases (Erler et al., 2009; Eisinger-Mathason et al., 2013; Schutze et al., 2015). Thus, targeting Hif-1α could improve response to therapy, and several approaches to inhibit Hif-1α signaling are at various stages of development (reviewed in Hu et al., 2013). As a transcription factor acting by protein–protein and protein–DNA interaction, Hif-1α is notoriously difficult to target. Antisense siRNA and shRNA strategies are mainly used to target Hif-1α in preclinical models: in prostate xenografts, a nanocarrier approach to deliver Hif-1α siRNA improved the efficacy of DOX treatment in rats (Liu X.Q. et al., 2012). In addition, in prostate xenografts, the effective downregulation of Hif-1α using *salmonella* to deliver shRNA-expressing plasmids resulted in increased sensitivity to cisplatin (CDDP) (Gu et al., 2017). However, for the improved drug distribution and response after either TGFβ- or Hif-1α targeting, alternative mechanisms cannot be excluded: TGFβ does not only induce increased ECM build-up and stabilization, but it is also an important regulator of tumor angiogenesis (reviewed in Goumans et al., 2009; van Meeteren et al., 2011). In the treatment study of 4T1 breast carcinomas with TGFβ inhibitors, Liu et al. observed not only a reduction in Col I deposition but also increased vessel maturation and improved vascular perfusion (Liu J. et al., 2012). Similarly, Hif-1α induces expression of the angiogenic factors VEGF-A, FGF-2, and SDF1 (Enholm et al., 1997; Tang et al., 2004; Du et al., 2008). Moreover, as outlined above, hypoxia and subsequent Hif-1α stabilization by itself upregulates various pathways mediating tumor cell resistance to chemotherapy and radiation (Sullivan et al., 2008; Kolenda et al., 2011). However, killing two birds with one stone is far from an undesirable, and Hif-1α targeting has the potential to simultaneously reduce metastatic and invasive behavior, sensitize tumor cells to therapy, and ameliorate the detrimental effects of a pathologically altered ECM and dysfunctional vasculature on drug supply, distribution, and resistance.

### Targeting CAFs

As mentioned before, stromal cells are a major source of the vast amounts of ECM that, in many tumors, obstruct efficient and homogeneous drug delivery. The infiltration and often encapsulation of tumors with CAFs parallels in many respects fibrosis under other pathological conditions. Therefore, it is of course evident to test antifibrotic drugs that are in clinical use for other ailments for their potential to reduce the malignant tumor ECM and to increase drug transport. Konzono et al. found that the efficacy of gemcitabine in pancreas cancer xenografts was increased after treatment with the antifibrotic drug pirfenidone (Kozono et al., 2013). However, pirfenidone had a significant antitumor effect by itself. Thus, it is possible that the observed increased efficacy of the combination only reflects an additive effect. In a trimodal combination approach, pirfenidone improved response to radiation and sunitinib in a murine Lewis lung carcinoma model (Choi et al., 2015). Histological analysis indicated that the increased collagen production caused by radiation reduced efficacy of sunitinib. This desmoplastic response was prevented by pirfenidone, restoring sunitinib sensitivity. Pirfenidone might have additional beneficial effects in the treatment of cancer: DOX has significant renal and cardiac toxicity limiting its lifetime doses in cancer patients. Pirfenidone ameliorated these toxic effects in rats, presumably by reducing the fibrotic reaction in the affected organs (Giri et al., 2004). Pirfenidone has also some sensitizing effect in 2D coculture experiments, indicating that it is not only improving therapy response by interfering with ECM production: by treating CAF-NSCLC cell cocultures with pirfenidone, the sensitivity of both cells was increased (Mediavilla-Varela et al., 2016). Ormeloxifene, an estrogen receptor modulator, in contrast to pirfenidone, had no substantial antitumor effect in the PDAC xenografts it was tested in Khan et al. (2015). Still, it improved the efficacy of gemcitabine treatment. Ormeloxifene also inhibits sonic hedgehog signaling, a pathway involved in desmoplasia in PDAC and reduced fibroblast infiltration and Col I deposition. Losartan, an angiotensin receptor antagonist, increased perfused vessel density and reduced Col I and hyaluronan synthesis in various murine tumors. This resulted in improved delivery and efficacy of 5-FU and DOX (Diop-Frimpong et al., 2011; Chauhan et al., 2013). Losartan also improved response to liposomal paclitaxel in a murine model for stage IV metastatic breast cancer (Zhang L. et al., 2016). This study also showed that losartan reduced TGFβ1 expression and subsequently the production of Col I and LOX.

### Clinical Trials

Considering the interesting results already obtained in preclinical tests, it is not surprising that several of the approaches to alter the ECM to improve response to concomitant therapy are already in at least early clinical test phases (see Table 3 for details).

**Table 3:** ECM-targeted drugs in combination with tumor-directed therapy in clinical trials.

**Target**	**Drug**	**Therapy used in combination with ECM-targeted drug**	**Malignancy**	**Trial phase**	**Status**	**References**
LOXL2	Simtuzumab	Gemcitabine	PDAC	II	Completed	Benson et al., 2017
		FOLFIRI^a^	Metastatic CRC	II	Completed	Hecht et al., 2017
HA	PEGPH20	Gemcitabine + nab-paclitaxel	Metastatic pancreatic cancer	II	Completed	^4^
		Gemcitabine	Stage IV pancreatic cancer	II	Completed	^5^
		Eribulin mesilate	Metastatic breast cancer	Ib	Active	^5^
		Pembrolizumab^c^	• NSCLC • Gastric cancer hyaluronan-high	Ib	Active	^6^
		Cetuximab^d^	Pancreatic cancer	I/II	Completed	^7^
		Avelumab^e^	Pancreatic cancer	I	Recruiting	^8^
		Gemcitabine + nab-paclitaxel^f^	Advanced pancreatic ductal adenocarcinoma	NA	Recruiting	^4^
		Atezolizumab^g^	Pancreatic adenocarcinoma	I/II	Recruiting	^9^
		Atezolizumab^g^	Gastric adenocarcinoma or gastroesophageal junction adenocarcinoma	I/II	Recruiting	^10^
		Cisplatin + Gemcitabine	Cholangiocarcinoma	I	Active	^11^
		Gemcitabine + nab-paclitaxel	PDAC	II	Recruiting	^12^
		Gemcitabine + nab-paclitaxel	PDAC	III	Active	^6^
Fibrosis	Pirfenidone	Carboplatin + Pemetrexed, Carboplatin + Paclitaxel	NSCLC	I	Recruiting	^7^
	Losartan	FOLFIRINOX^b^, Nivolumab^h^, Radiation therapy	Pancreatic cancer	II	Recruiting	^8^
		FOLFIRINOX^b^ + proton beam radiation	Pancreatic cancer	II	Active	^9^
		Sunitinib	Osteosarcoma	I	Announced	^10^
TGFβ	Galunisertib (Ly2157299)	Carboplatin + paclitaxel	Ovarian and uterus carcinoma	I	Recruiting	^13^
		Radiotherapy	Metastatic breast cancer	II	Active	^14^
		Nivolumab	NSCLC, hepatocellular cancer	I/II	Active	^15^
	Fresolimumab	Radiotherapy	NSCLC	I/II	Recruiting	^16^

a *FOLFIRI: Folinic acid (leucovorin), 5-FU, irinotecan*.

b *FOLFIRINOX: Folinic acid (leucovorin), 5-FU, irinotecan, oxaliplatin*.

c *Pembrolizumab: anti-PD-1*.

d *Cetuximab: anti-EGFR*.

e *Avelumab: anti-PD-L1*.

f *nab-paclitaxel: albumin bound paclitaxel*.

g *Atezolizumab: anti-PD-L1*.

h *Nivolumab: anti-PD-1*.

We already mentioned the completed phase II trials using LOXL2 antibody in combination with gemcitabine and FOLFIRI in PDAC and CRC patients, respectively (Benson et al., 2017; Hecht et al., 2017). The furthest progress was probably the clinical evaluation of hyaluronidase as an auxiliary treatment. Pegylated hyaluronidase (PEGPH20) is tested in PDAC in combination with gemcitabine, nab-paclitaxel, avelumab, and cetuximab (Hingorani et al., 2016; Doherty et al., 2018; Gourd, 2018; Infante et al., 2018)^17^, in breast cancer with erlotinib^18^ and a range of other drugs in various different cancers. As far as the studies are completed and evaluated, the results appear more encouraging than those from the LOXL2 trials, and a phase III trial combining PEGPH20 with nab-paclitaxel plus gemcitabine in PDAC started already in early 2016^19^. Pirfenidone plus standard of care chemotherapy is undergoing phase I evaluation in NSCLC^20^. In addition, pirfenidone is tested for its ability to ameliorate fibrosis in cancer patients resulting from radiotherapy. As mentioned above, the angiotensin-II inhibitor losartan might, in addition to its vascular effects, considerably affect the ECM. It is under clinical investigation in combination with immuno-, radio-, and chemotherapy^21^^,^ ^22^^,^^23^.

## Concluding Remarks

The ECM in solid tumors is strongly involved in determining the course of the disease and also the results of our efforts to treat malignancies. Interfering with the synthesis and accumulation of ECM or related processes has the potential to significantly improve the outcome of concomitant therapeutic approaches, whether these are conventional cytotoxic treatments, radiotherapy, or targeted therapy including immunotherapy. We have already begun to explore the potential of ECM targeting to improve response to antineoplastic therapy. These research efforts are still mainly in the realms of basic and early translational research and often far from reaching the clinic. However, there are major challenges we will face over the next decades. First, from the view of basic scientist interested in understanding the fundamental mechanisms behind how the ECM influences cancer treatment, there is the problem that targeting ECM synthesis almost always affects other processes in the TME. Likewise, targeting other components of the TME, e.g., angiogenesis or immune cells also affects the ECM. This mutual interdependence of various processes in the tumor often complicates the interpretation of experiments. A meticulous understanding of the underlying mechanisms, however, is often necessary for successful translation in the clinic. Second, the ECM is a complex mixture of numerous macromolecules, with divers characteristics, that often rely on complicated multistep processes for synthesis. This enables interfering with tumoral ECM accumulation in various ways and opens the possibility for tailor-made ECM-targeting strategies for various malignancies. However, it is also a challenge to select from this multitude of possible points of attack, the right ones, that promise the strongest effects and that are least likely cause problems with the functions of the ECM in physiological processes. Finally, although ECM destabilization can already positively affect the course of neoplastic diseases, by reducing invasiveness, progression, and metastasis, the most likely application for ECM-targeted approaches is not a stand-alone therapy but modalities where they are used to cause synergistic improvements in combination with other tumor-directed forms of therapy. Combining various forms that are supposed to synergistically improve each other's performance asks for successively more complicated forms of clinical evaluation, with more extensive planning, more detailed follow-up procedures, more surrogate endpoints, and possibly more trial arms. If we master these challenges, ECM-targeting approaches have the potential to significantly add to our repertoire of cancer-fighting strategies and moreover strongly improve the performance of the therapies already at our hand.

## Author Contributions

EH, RN, and SE wrote the manuscript.

### Conflict of Interest

The authors declare that the research was conducted in the absence of any commercial or financial relationships that could be construed as a potential conflict of interest.

## Footnotes

^1^*FDA Approves Keytruda (pembrolizumab) for Advanced Melanoma*. Available online at: https://www.drugs.com/newdrugs/fda-approves-keytruda-pembrolizumab-advanced-melanoma-4079.html (accessed December 22, 2018).

^2^*FDA Approves YERVOY*™ *(ipilimumab) for the Treatment of Patients with Newly Diagnosed or Previously-Treated Unresectable or Metastatic Melanoma, the Deadliest form of Skin Cancer*. Available online at: https://news.bms.com/press-release/rd-news/fda-approves-yervoy-ipilimumab-treatment-patients-newly-diagnosed-or-previousl.

^3^*FDA Grants Accelerated Approval to Ipilimumab for MSI-H or dMMR Metastatic Colorectal Cance*. Available online at: https://www.fda.gov/drugs/informationondrugs/approveddrugs/ucm613227.htm.

^4^*PEGPH20 Plus Nab-Paclitaxel Plus Gemcitabine Compared with Nab-Paclitaxel Plus Gemcitabine in Subjects with Stage IV Untreated Pancreatic Cancer (HALO-109-202)*. Available online at: https://clinicaltrials.gov/ct2/show/record/NCT01839487 (accessed May 2, 2019).

^5^*Study of Gemcitabine* + *PEGPH20 vs Gemcitabine Alone in Stage IV Previously Untreated Pancreatic Cancer*. Available online at: https://clinicaltrials.gov/ct2/show/NCT01453153 (accessed May 2, 2019).

^6^*Phase 1b Open-Label Study of PEGylated Recombinant Human Hyaluronidase (PEGPH20) with Pembrolizumab*. Available online at: https://clinicaltrials.gov/ct2/show/NCT02563548 (accessed May 2, 2019).

^7^*Two Stage Study of Single Dose PEGPH20 and Cetuximab in Patients with Pancreatic Adenocarcinoma Prior to Surgical Resection*. Available online at: https://clinicaltrials.gov/ct2/show/NCT02241187 (accessed May 2, 2019).

^8^*A Trial of PEGPH20 in Combination with Avelumab in Chemotherapy Resistant Pancreatic Cancer*. Available online at: https://clinicaltrials.gov/ct2/show/NCT03481920 (accessed May 2, 2019).

^9^*A Study of Multiple Immunotherapy-Based Treatment Combinations in Participants with Metastatic Pancreatic Ductal Adenocarcinoma (Morpheus-Pancreatic Cancer)*. Available online at: https://clinicaltrials.gov/ct2/show/NCT03193190 (accessed May 2, 2019).

^10^*A Study of Multiple Immunotherapy-Based Treatment Combinations in Patients with Locally Advanced Unresectable or Metastatic Gastric or Gastroesophageal Junction Cancer (G/GEJ) (Morpheus-Gastric Cancer)*. Available online at: https://clinicaltrials.gov/ct2/show/NCT03281369 (accessed May 2, 2019).

^11^*Study of PEGPH20 with Cisplatin (CIS) and Gemcitabine (GEM); PEGPH20 with Atezolizumab, CIS, and GEM; and CIS and GEM Alone in Participants with Previously Untreated, Unresectable, Locally Advanced, or Metastatic Intrahepatic and Extrahepatic Cholangiocarcinoma and Gallbladder Adenocarcinoma*. Available online at: https://clinicaltrials.gov/ct2/show/NCT03267940 (accessed May 2, 2019).

^12^*PEGPH20, Gemicitabine and Nab-Paclitaxel for Pancreatic Ductal Adenocarcinoma*. Available online at: https://clinicaltrials.gov/ct2/show/NCT02487277 (accessed May 2, 2019).

^13^*Paclitaxel/Carboplatin* + *Galunisertib for Patients with Carcinosarcoma of the Uterus or Ovary*. Available online at: https://clinicaltrials.gov/ct2/show/NCT03206177 (accessed May 2, 2019).

^14^*LY2157299 Monohydrate (LY2157299) and Radiotherapy in Metastatic Breast Cancer*. Available online at: https://clinicaltrials.gov/ct2/show/NCT02538471 (accessed May 2, 2019).

^15^*A Study of Galunisertib (LY2157299) in Combination with Nivolumab in Advanced Refractory Solid Tumors and in Recurrent or Refractory NSCLC, or Hepatocellular Carcinoma*. Available online at: https://clinicaltrials.gov/ct2/show/NCT02423343 (accessed May 2, 2019).

^16^*SABR-ATAC: A Trial of TGF-beta Inhibition and Stereotactic Ablative Radiotherapy for Early Stage Non-small Cell Lung Cancer*. Available online at: https://clinicaltrials.gov/ct2/show/NCT02581787 (accessed May 2, 2019).

^17^Study of Gemcitabine, Nab-paclitaxel, PEGPH20 and Rivaroxaban for Advanced Pancreatic Adenocarcinoma. Available online at: https://clinicaltrials.gov/ct2/show/NCT02921022 (accessed May 2, 2019).

^18^Study of Eribulin Mesylate in Combination with PEGylated Recombinant Human Hyaluronidase (PEGPH20) Versus Eribulin Mesylate Alone in Subjects with Human Epidermal Growth Factor Receptor 2 (HER2)-Negative, High-Hyaluronan (HA) Metastatic Breast Cancer (MBC). Available online at: https://clinicaltrials.gov/ct2/show/NCT02753595 (accessed May 2, 2019).

^19^*A Study of PEGylated Recombinant Human Hyaluronidase in Combination with Nab-Paclitaxel Plus Gemcitabine Compared with Placebo Plus Nab-Paclitaxel and Gemcitabine in Participants with Hyaluronan-High Stage IV Previously Untreated Pancreatic Ductal Adenocarcinoma*. Available online at: https://clinicaltrials.gov/ct2/show/NCT02715804 (accessed May 2, 2019).

^20^*Pirfenidone Combined with Standard First-Line Chemotherapy in Advanced-Stage Lung NSCLC*. Available online at: https://clinicaltrials.gov/ct2/show/NCT03177291 (accessed May 2, 2019).

^21^*Losartan and Nivolumab in Combination with FOLFIRINOX and SBRT in Localized Pancreatic Cancer*. Available online at: https://clinicaltrials.gov/ct2/show/NCT03563248 (accessed May 2, 2019).

^22^*Proton w/FOLFIRINOX-Losartan for Pancreatic Cancer*. Available online at: https://clinicaltrials.gov/ct2/show/NCT01821729 (accessed May 2, 2019).

^23^*Losartan* + *Sunitinib in Treatment of Osteosarcoma*. Available online at: https://clinicaltrials.gov/ct2/show/NCT03900793 (accessed May 2, 2019).
